# A novel nomogram for survival prediction in renal cell carcinoma patients with brain metastases: an analysis of the SEER database

**DOI:** 10.3389/fimmu.2025.1572580

**Published:** 2025-06-30

**Authors:** Fei Wang, Xihao Wang, Zhigang Feng, Jun Li, Hailiang Xu, Hengming Lu, Lianqu Wang, Zhihui Li

**Affiliations:** ^1^ Department of Reproductive Medicine, Central Hospital of Zhumadian, Henan, China; ^2^ Department of Urology, The First Affiliated Hospital of Henan University, Kaifeng, China; ^3^ Department of Urology, Central Hospital of Zhumadian, Henan, China; ^4^ Department of Urology, Women and Children’s Hospital, Central Hospital of Zhumadian, Henan, China; ^5^ Department of Gastroenterology, Central Hospital of Zhumadian, Henan, China

**Keywords:** renal cell carcinoma, brain metastases, nomogram, surgery, SEER

## Abstract

**Background:**

Existing research on the development of prognostic models for renal cell carcinoma (RCC) patients with brain metastases (BM) remains limited. This study aimed to develop a prognostic prediction model for RCC patients with BM and to identify critical factors influencing clinical outcomes.

**Methods:**

Patients diagnosed with BM between 2010 and 2019 were identified and extracted from the Surveillance, Epidemiology, and End Results (SEER) database. Potential risk factors were initially screened applying the eXtreme Gradient Boosting (XGBoost) and Random Forest (RF) machine learning algorithms. Subsequently, multivariate COX regression analysis was performed to identify independent risk factors for constructing the predictive nomogram. Nomogram performance was comprehensively evaluated based on Harrell’s concordance index (C-index), receiver operating characteristic (ROC) curve analysis, calibration plots, and decision curve analysis (DCA). The SHapley Additive exPlanations (SHAP) method was employed to demonstrate the ranking of feature importance affecting patient prognosis at different time points. Moreover, we conducted propensity score matching (PSM) and Kaplan-Meier (K-M) survival analysis to compare clinical outcomes between surgical and non-surgical treatment subgroups.

**Results:**

In total, 982 patients were assigned to the training cohort and 420 to the validation cohort. The constructed nomogram included four clinical variables: histologic type, T stage, N stage, surgery and chemotherapy. The AUC, C-index, calibration curves, and DCA curves showed excellent performance of the nomogram. In addition, the SHAP values indicated that surgical treatment was the most important prognostic risk factor for OS at 6-months, 1-year, 2-years, and 3-years. After further balancing the baseline characteristics between the surgical and non-surgical groups using PSM, we observed that patients with BM who underwent surgical intervention showed significantly better survival outcomes across all subgroups compared to non-surgical patients, though unmeasured confounders may contribute to this association.

**Conclusion:**

We developed a novel nomogram for predicting prognostic factors in RCC patients with BM, offering a valuable tool to support accurate clinical decision-making. Our research also confirmed that surgical intervention was significantly associated with improved survival outcomes for patients with BM.

## 1 Introduction

Over the past two decades, the global incidence of renal cell carcinoma (RCC) has shown a persistent upward trend, now representing approximately 3% of all newly diagnosed malignancies (1). The disease burden is substantial, with 431,288 new cases and 179,368 deaths reported worldwide in 2020 alone (2). The three major histopathological subtypes of RCC are clear cell carcinoma (ccRCC), papillary carcinoma (pRCC), and chromophobe renal cell carcinoma (chrRCC). Studies have reported that ccRCC has the poorest survival rate among these subtypes (3, 4). In addition to primary renal lesions, metastatic lesions may occur at other sites, which are referred to as metastatic renal cell carcinoma (mRCC). Metastatic progression remains a critical challenge, as evidenced by the fact that one-third of patients already present with mRCC at initial diagnosis (5).

The brain is one of the most common sites for metastatic spread in malignant tumors. Nearly 10% of cancer patients will develop brain metastases (BM) at some point during the course of their disease, and approximately 10-26% of cancer-related deaths are attributable to BM (6, 7). A study based on data from the International Metastatic Renal Cell Carcinoma Database Consortium (IMDC) found that 8.1% of patients with advanced RCC had BM at the time of initiating systemic treatment, and these patients had a significantly worse prognosis compared to those without BM (8). A multicenter retrospective study involving 226 patients with histologically diagnosed RCC and radiographic evidence of BM revealed a median overall survival (OS) of 18.8 months (interquartile range: 6.2–43 months) (9). Additionally, a retrospective review demonstrated that among 72 patients with asymptomatic BM from mRCC, 38.5% had multifocal central nervous system involvement, 40% had brain tumors larger than 1 cm in diameter, and their median OS was only 10.3 months (10). Such findings underscore BM as an independent predictor of mortality in RCC, highlighting the urgent need for reliable prognostic tools to guide clinical management.

In recent years, the nomogram has emerged as a widely utilized tool in oncological prognostic studies, exhibiting exceptional predictive performance across diverse cancer types (11–14). As a comprehensive predictive model, the nomogram represents a significant advancement in personalized medicine, offering clinicians an effective and user-friendly instrument for evaluating cancer prognosis and facilitating individualized treatment strategies (15). Given the low incidence of BM in RCC patients, well-established prognostic models specifically tailored for this patient population remain scarce in clinical practice. Although Zhuang et al. (16) developed a nomogram for RCC patients with BM using the Surveillance, Epidemiology, and End Results (SEER) data, limitations such as loose inclusion criteria, high missing data rates, and lack of external validation hinder its clinical utility. A more robust and generalizable prognostic tool is urgently needed to guide personalized management.

To address these gaps, we analyzed SEER data from 2010 to 2019 and proposed an innovative approach integrating eXtreme Gradient Boosting (XGBoost) and Random Forest (RF) algorithms to identify key prognostic variables. Subsequently, a multivariate Cox regression-based nomogram was constructed and rigorously validated. Furthermore, to enhance interpretability, SHapley Additive exPlanations (SHAP) values were utilized to quantify feature importance, while propensity score matching (PSM) was employed to evaluate the survival benefits associated with surgical intervention.

In this paper, our study addresses two pivotal research questions: (1) what are the independent prognostic factors influencing overall survival in RCC patients with BM? (2) Does surgical intervention confer a significant survival benefit in this patient population? Our findings aim to provide evidence-based support for clinical decision-making.

## 2 Materials and methods

### 2.1 Data acquisition and data extraction

This retrospective study utilized data from the National Cancer Institute’s SEER 17 Registries Database (Incidence-SEER Research Data, 17 Registries, Nov 2023 Sub [2000-2021], released April 2024, version 8.4.4). The institutional review board granted a waiver of informed consent as the study involved analysis of de-identified public surveillance data. All procedures were conducted in accordance with the ethical standards of the Declaration of Helsinki. The dataset included cases diagnosed between January 1, 2010, and December 31, 2019, extracted from the most recent SEER database update.

The SEER database initially yielded 2,068 patients diagnosed between 2010 and 2019. Eligibility criteria comprised: (1) histologically confirmed RCC (ICD-O-3 site code C64.9); (2) RCC was the patients’ only cancer that had been diagnosed; (3) all RCC patients showed histopathological evidence of the disease; (4) all RCC patients developed BM at the time of initial diagnosis. Exclusion criteria were: (1) age <18 years at diagnosis; (2) non-unilateral RCC; (3) incomplete records (demographics, surgical intervention, survival data). After applying these criteria, 1,402 patients constituted the final analytic cohort.

### 2.2 Variable extraction

The analysis incorporated a comprehensive set of risk factors, including: age at diagnosis, sex, race, marital status (categorized as married, single, or divorced/separated/widowed [DSW]), histological subtype [ccRCC), pRCC and others], tumor laterality, tumor grade (ranging from well-differentiated [I] to undifferentiated [IV], with an additional category for unknown grade), surgical intervention status, tumor size (defined as the maximum diameter of the primary renal tumor, measured in millimeters), chemotherapy and radiotherapy status, presence of distant metastases (in bone, liver, or lung), time interval from diagnosis to treatment initiation, survival status, and survival time. Furthermore, tumor staging was conducted according to the AJCC classification system, with T-stage categorized as T1 through T4 and N-stage as N0 or N1.

### 2.3 Handling missing values

**Figure 1:**
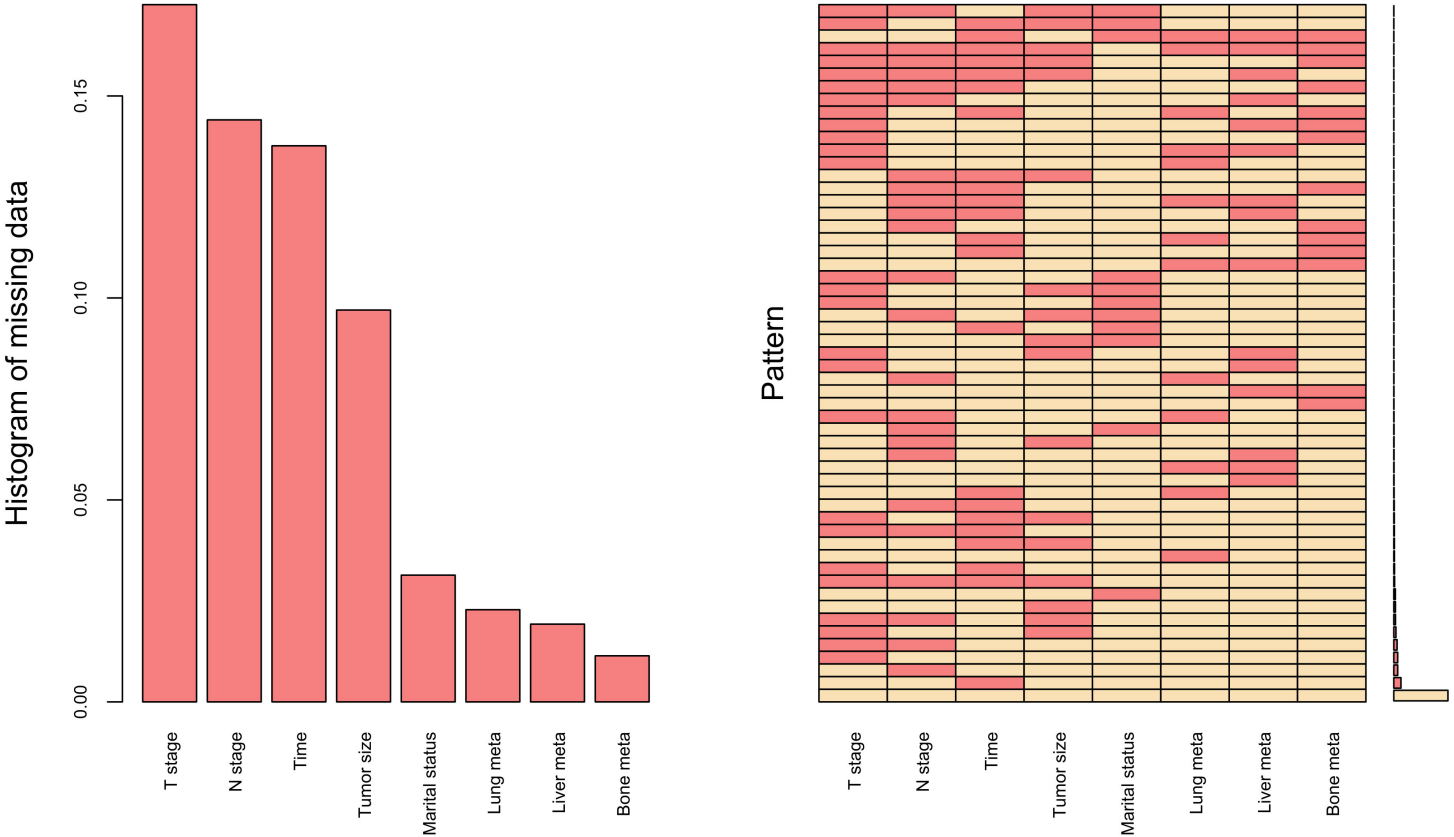
Missing data for research variables, including T stage, N stage, Time (time from diagnosis to therapy), Tumor size, Marital status, Lung meta (lung metastases), Liver meta (liver metastases) and Bone meta (bone metastases).

Missing data represents a prevalent challenge in clinical research, where the common practice of simply deleting incomplete cases may result in significant information loss and inefficient utilization of valuable resources. To address this issue, data imputation emerges as a more scientifically sound and methodologically robust approach. **Figure 1** presents a comprehensive visualization of the missing data patterns observed in our study dataset. Moving beyond conventional imputation techniques, such as multiple imputation and median imputation, we implemented the K-nearest neighbors (KNN) imputation method, which has been extensively validated and showed superior performance in numerous studies (16, 17). Our implementation specifically utilized the VIM R package (hyperparameters: k=10, meth=“most”) to handle variables with limited missing data. To ensure methodological rigor, we partitioned the dataset into training and testing subsets using a 7:3 ratio, thereby facilitating robust model development and validation.

### 2.4 Selection of prognostic feature variables

Identifying risk factors associated with tumor prognosis was challenging, as traditional statistical methods might not always have yielded satisfactory results. As a result, researchers turned to ML algorithms to identify potential risk variables, achieving promising predictive outcomes (18–20). In the training set, we employed both XGBoost and RF algorithms to select variables related to the prognosis of RCC patients with BM. The intersection of variables selected by both algorithms was visualized through a Venn diagram, ensuring robust feature selection. Subsequently, multivariable Cox proportional hazards regression was performed on these consensus variables to identify independent predictors of OS for patients with BM.

### 2.5 Nomogram model development and validation

Based on variables selected through multivariable regression analysis, we constructed a nomogram to predict the prognosis of RCC patients with BM, aiming to guide clinical decision-making. The model’s predictive performance was assessed using the concordance index (C-index), receiver operating characteristic (ROC) curves, calibration plots, and decision curve analysis (DCA). To rigorously evaluate model robustness, we performed 1000 bootstrap resampling replicates in addition to the initial 70:30 split. The mean C-index across bootstrap iterations was calculated to correct for overoptimism bias. Additionally, risk stratification was implemented based on the model, and survival differences among distinct risk subgroups were compared via Kaplan-Meier (KM) analysis.

### 2.6 Model explanation

The XGBoost, an algorithmic framework based on the Gradient Boosting Decision Tree (GBDT), has been widely utilized in tumor prognostic studies and has demonstrated promising predictive performance (21, 22). The SHAP represents a methodology rooted in cooperative game theory, designed to interpret black-box models such as ML systems (23). The SHAP approach enhances model transparency and interpretability by quantifying the contribution of each feature to prediction outcomes through both local instance-specific and global model-wide explanations (24). In our study, we selected the XGBoost model as our predictive framework and systematically ranked the importance of features influencing prognostic risk factors in descending order for RCC patients with BM.

### 2.7 Statistical analysis

We performed data analysis and model development using R (version 4.4.1; R Foundation for Statistical Computing) and Python (version 3.11.5; Python Software Foundation). To evaluate potential differences in baseline characteristics between the training and validation cohorts, we applied the Mann-Whitney U test for non-normally distributed continuous variables and the Chi-squared test for categorical variables. PSM was implemented to balance baseline characteristics between the non-surgical group and the radical nephrectomy group. We subsequently investigated the differences in survival outcomes, specifically OS and cancer-specific survival (CSS), between the surgical and non-surgical groups across various subgroups. All statistical tests were two-tailed, and a significance threshold of P < 0.05 was adopted.

## 3 Results

### 3.1 Baseline characteristics of included patients

A total of 1,402 RCC patients with BM (68.05% male and 31.95% female) were included in the derivation dataset, with a median age of 61.00 years. Among them, 1,205 (85.95%) were White, 833 (59.42%) were married, and 982 (70.04%) had a median annual household income between $50000 to $89999. A total of 696 patients (49.64%) had ccRCC. Regarding tumor grade, 27 patients (1.93%) were grade I, 129 (9.20%) were grade II, 237 (16.90%) were grade III, and 164 (11.70%) were grade IV. The tumor was located on the left side in 708 patients (50.50%). Additionally, 359 patients (25.61%) underwent surgery, 1,035 (73.82%) received radiotherapy, and 681 (48.57%) received chemotherapy. The median tumor diameter was 90.00 mm. Tumor stages were distributed as follows: T1 in 309 patients (22.04%), T2 in 454 (32.38%), T3 in 489 (34.88%), and T4 in 150 (10.70%). Among the patients, 979 (69.83%) had N0 stage. A total of 579 patients (41.30%) had bone metastases, 270 (19.26%) had liver metastases, and 1,002 (71.47%) had lung metastases. The time from diagnosis to treatment was less than one month for 1,104 patients (78.74%). The median follow-up time was 5 months. No significant statistical differences were observed between the training and validation cohorts except for median age. **Table 1** summarizes the baseline characteristics of patients with BM in the training and validation cohorts.

**Table 1:** Demographic and clinicopathological characteristics for RCC patients with BM.

Characteristics	Total (N = 1,402)	Training cohort (N = 982)	Validation cohort (N = 420)	P-value
Age (years)	61.00 (55.00, 68.00)	61.00 (54.00, 67.00)	62.50 (55.00, 69.00)	0.021
Age group (years)				0.217
<40	26 (1.85%)	22 (2.24%)	4 (0.95%)	
40-49	139 (9.91%)	102 (10.39%)	37 (8.81%)	
50-59	429 (30.60%)	308 (31.36%)	121 (28.81%)	
60-69	513 (36.59%)	357 (36.35%)	156 (37.14%)	
70-79	235 (16.76%)	154 (15.68%)	81 (19.29%)	
80+	60 (4.28%)	39 (3.97%)	21 (5.00%)	
Sex				0.971
Male	954 (68.05%)	669 (68.13%)	285 (67.86%)	
Female	448 (31.95%)	313 (31.87%)	135 (32.14%)	
Race				0.543
White	1,205 (85.95%)	841 (85.64%)	364 (86.67%)	
Black	92 (6.56%)	69 (7.03%)	23 (5.48%)	
Other	105 (7.49%)	72 (7.33%)	33 (7.86%)	
Marital status				0.652
Married	833 (59.42%)	583 (59.37%)	250 (59.52%)	
Single	288 (20.54%)	207 (21.08%)	81 (19.29%)	
D/S/W	281 (20.04%)	192 (19.55%)	89 (21.19%)	
Histologic type				0.800
ccRCC	696 (49.64%)	482 (49.08%)	214 (50.95%)	
pRCC	36 (2.57%)	25 (2.55%)	11 (2.62%)	
Other	670 (47.79%)	475 (48.37%)	195 (46.43%)	
Grade				0.484
I	27 (1.93%)	18 (1.83%)	9 (2.14%)	
II	129 (9.20%)	84 (8.55%)	45 (10.71%)	
III	237 (16.90%)	173 (17.62%)	64 (15.24%)	
IV	164 (11.70%)	110 (11.20%)	54 (12.86%)	
Unknown	845 (60.27%)	597 (60.79%)	248 (59.05%)	
Laterality				0.388
Left	708 (50.50%)	488 (49.69%)	220 (52.38%)	
Right	694 (49.50%)	494 (50.31%)	200 (47.62%)	
T stage				0.816
T1	309 (22.04%)	217 (22.10%)	92 (21.90%)	
T2	454 (32.38%)	316 (32.18%)	138 (32.86%)	
T3	489 (34.88%)	339 (34.52%)	150 (35.71%)	
T4	150 (10.70%)	110 (11.20%)	40 (9.52%)	
N stage				0.877
N0	979 (69.83%)	684 (69.65%)	295 (70.24%)	
N1	423 (30.17%)	298 (30.35%)	125 (29.76%)	
Surgery				0.184
No	1,043 (74.39%)	741 (75.46%)	302 (71.90%)	
Yes	359 (25.61%)	241 (24.54%)	118 (28.10%)	
Radiation				0.648
No	367 (26.18%)	261 (26.58%)	106 (25.24%)	
Yes	1,035 (73.82%)	721 (73.42%)	314 (74.76%)	
Chemotherapy				0.381
No	721 (51.43%)	497 (50.61%)	224 (53.33%)	
Yes	681 (48.57%)	485 (49.39%)	196 (46.67%)	
Size (mm)	90.00 (68.00, 110.00)	90.00 (69.04, 111.00)	87.50 (65.00, 108.25)	0.078
Months from diagnosis to therapy				0.591
0 month	1,104 (78.74%)	769 (78.31%)	335 (79.76%)	
≥ 1 month	298 (21.26%)	213 (21.69%)	85 (20.24%)	
Bone metastases				0.154
No	823 (58.70%)	589 (59.98%)	234 (55.71%)	
Yes	579 (41.30%)	393 (40.02%)	186 (44.29%)	
Liver metastases				0.724
No	1,132 (80.74%)	790 (80.45%)	342 (81.43%)	
Yes	270 (19.26%)	192 (19.55%)	78 (18.57%)	
Lung metastases				0.111
No	400 (28.53%)	293 (29.84%)	107 (25.48%)	
Yes	1,002 (71.47%)	689 (70.16%)	313 (74.52%)	
Median household income				0.830
< 50,000$	116 (8.27%)	84 (8.55%)	32 (7.62%)	
50,000–69,999$	432 (30.81%)	296 (30.14%)	136 (32.38%)	
70,000–89,999$	550 (39.23%)	387 (39.41%)	163 (38.81%)	
90,000$ +	304 (21.68%)	215 (21.89%)	89 (21.19%)	
Survival time (months)	5.00 (2.00, 14.00)	5.00 (2.00, 15.00)	5.00 (2.00, 14.00)	0.868

D/S/W, divorced/separated/widowed; ccRCC, clear cell renal cell carcinoma; pRCC, papillary renal cell carcinoma.

### 3.2 Potential predictors of OS in patients with BM

**Figure 2:**
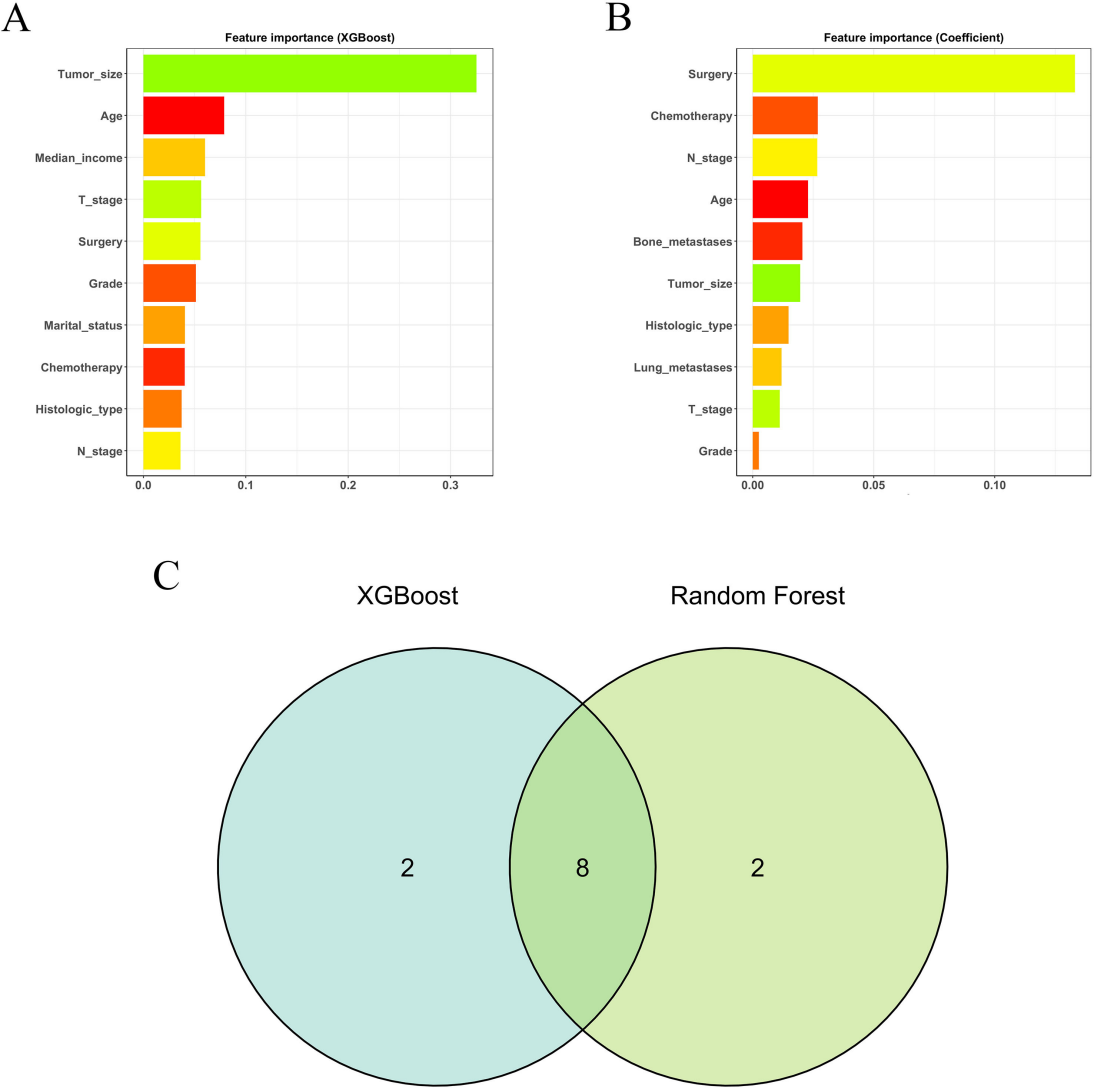
The results of XGBoost **(A)** and RF **(B)** machine learning algorithms filter the top 10 important variables. The results are expressed by coefficient value. **(C)** Venn analysis of the results of the above two machine algorithms.

**Figure 3:**
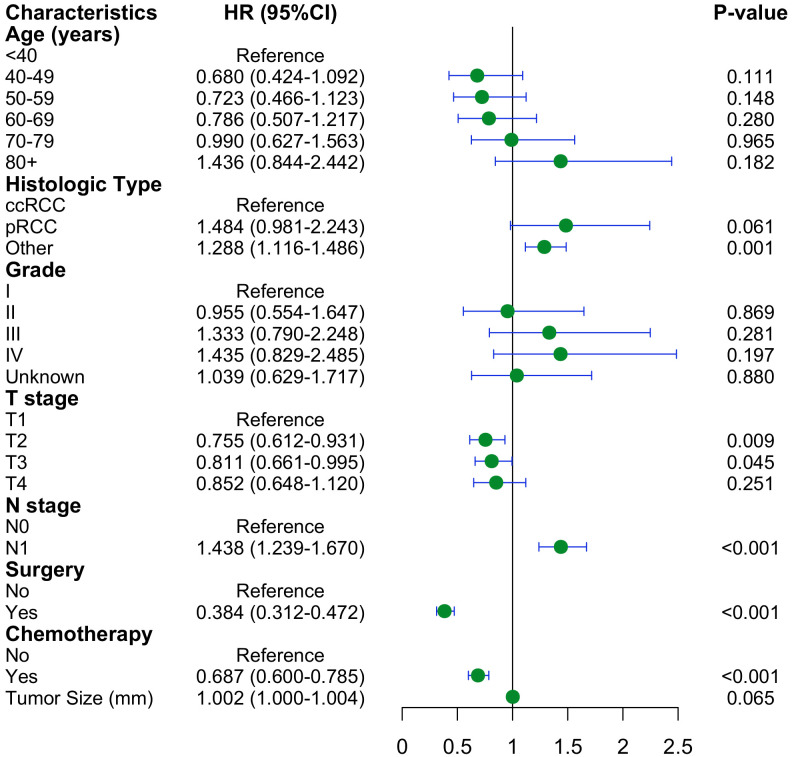
Forest plot with hazard ratios (HR) for the optimal prognostic variables of the multivariate Cox regression in the training cohort.

The preliminary screening of feature variables was performed using two ML algorithms, namely XGBoost (**Figure 2A**) and RF (**Figure 2B**). Both algorithms were employed to identify the top 10 most important variables in their respective models. A comprehensive analysis using a Venn diagram revealed 8 key variables: age, histologic type, grade, T stage, N stage, surgery, chemotherapy, and tumor size (**Figure 2C**). Subsequently, multivariable Cox regression analysis was performed to control for confounding factors and identify independent variables associated with OS (**Figure 3**). The analysis indicated that surgery, chemotherapy, and N stage were significantly associated with worse OS. Patients with histologic subtypes other than ccRCC exhibited worse OS compared to those with ccRCC. Unexpectedly, T2, T3 and T4 stages were associated with better OS than T1 stage, while no significant difference in OS was observed between T1 and T4 stages.

### 3.3 Construction and performance of the nomogram

**Figure 4:**
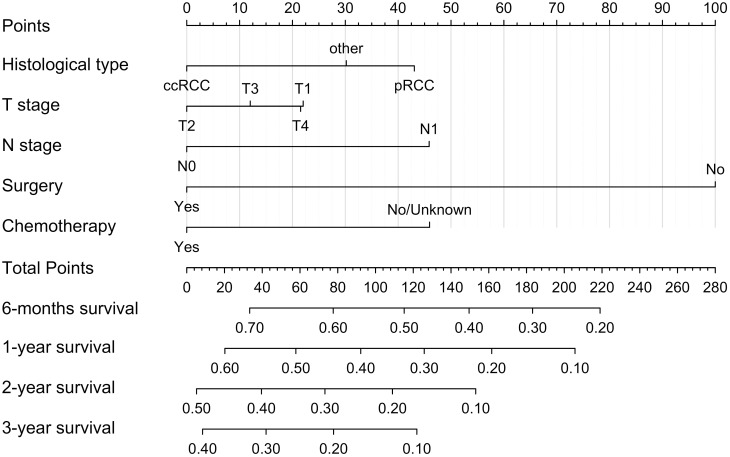
Nomogram for predicting 6-moths, 1-year, 2-year, and 3-year OS for RCC patients with BM in the training cohort.

**Figure 5:**
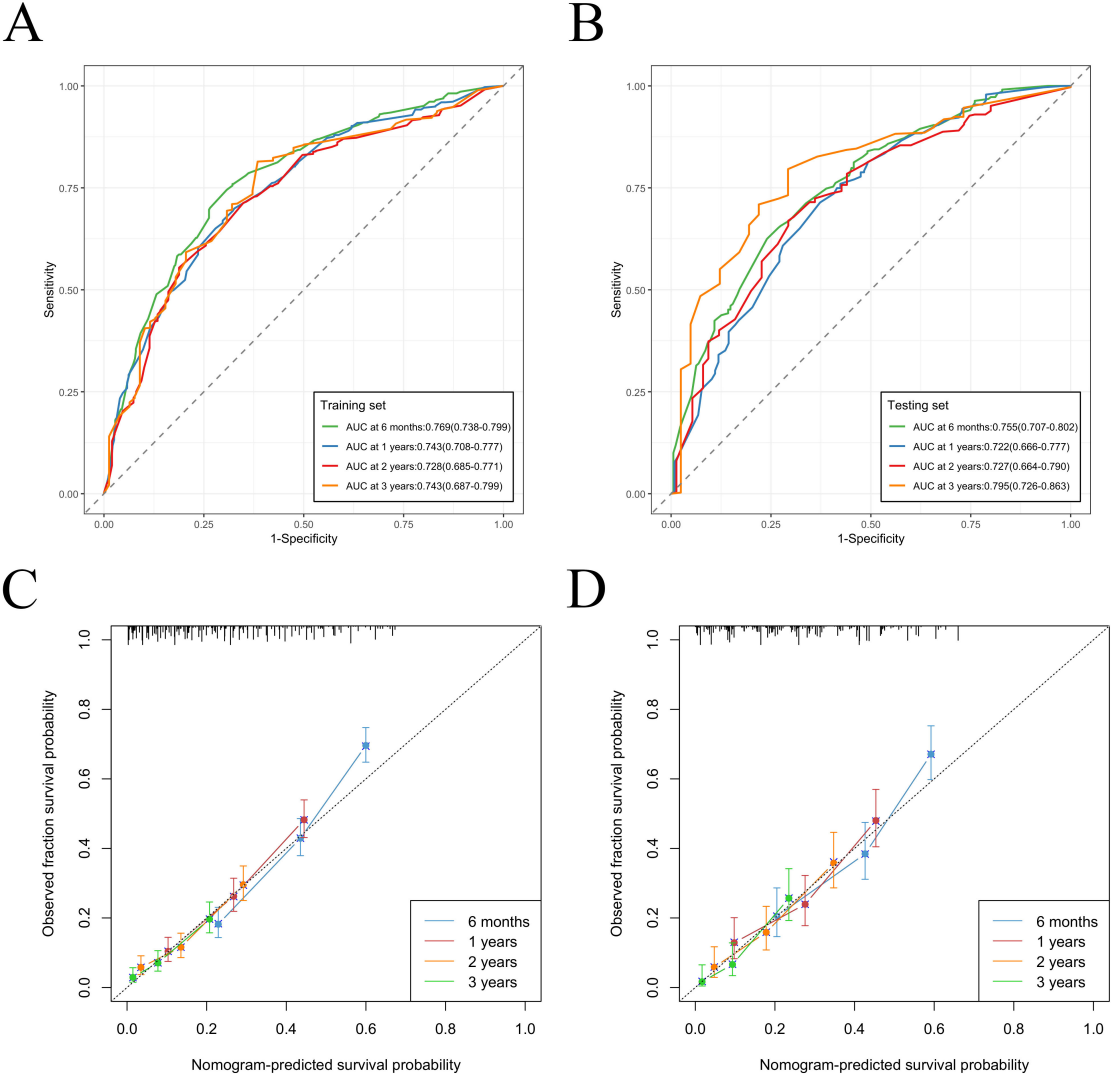
Nomogram ROC curves to predict 6-months 1-year, 2-year and 3-year OS in the training cohort **(A)** and in the validation cohort **(B)**. Nomogram calibration curves to predict 6-months 1-year, 2-year and 3-year OS in the training cohort **(C)** and in the validation cohort **(D)**.

**Figure 6:**
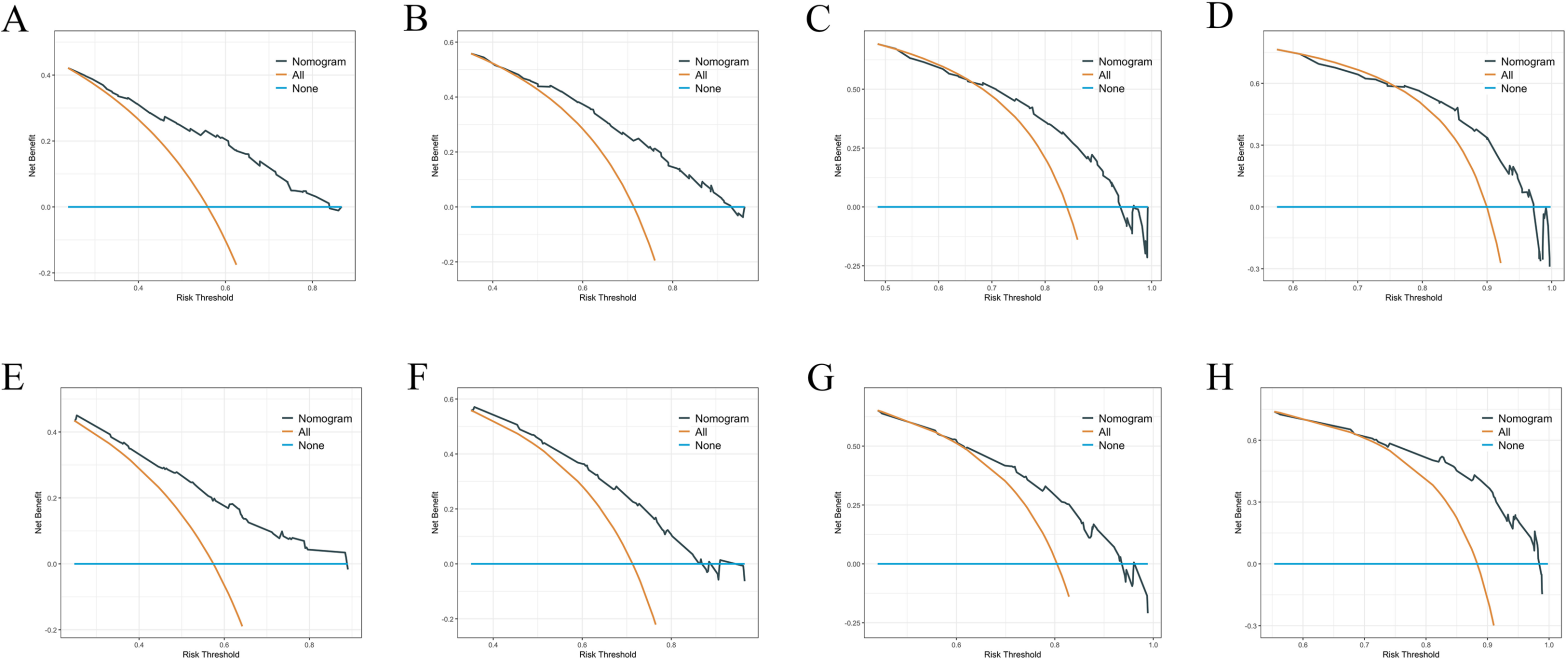
**(A–D)** DCA analysis predicting 6-months 1-year, 2-year and 3-year OS in the training cohort. **(E–H)** DCA analysis predicting 6-months 1-year, 2-year and 3-year OS in the validation cohort.

**Figure 7:**
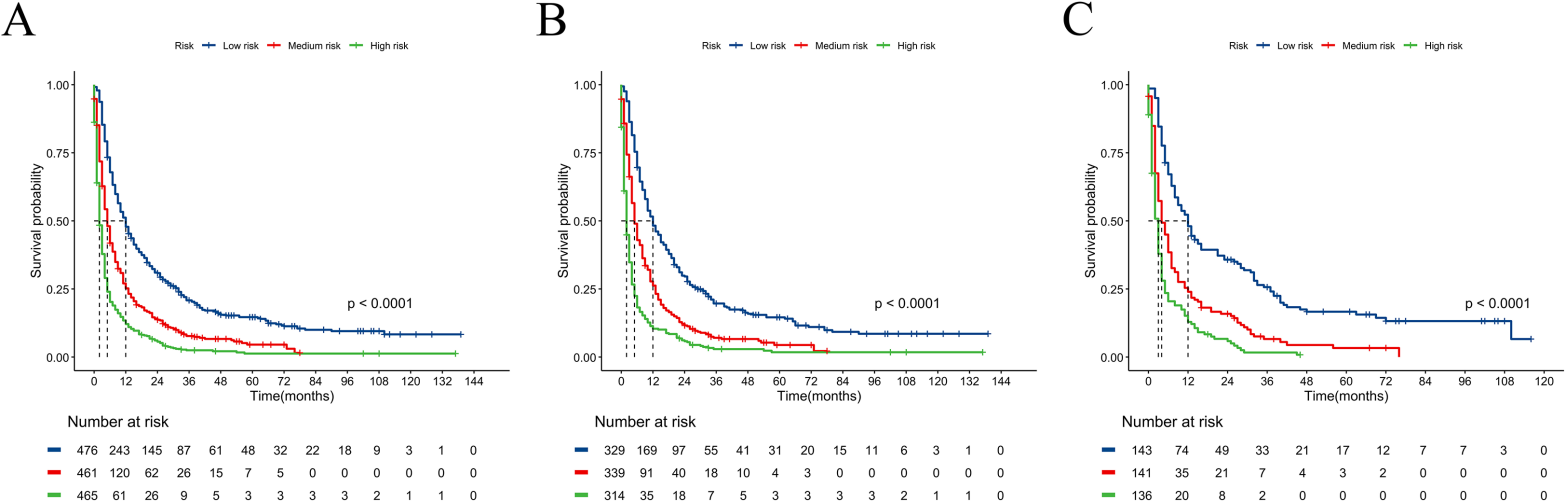
Kaplan–Meier curves for predicting OS of RCC patients with BM in low-risk, medium-risk, and high-risk groups. **(A)** For all cohort; **(B)** For training cohort; **(C)** For validation cohort.

We constructed a nomogram model based on 5 independent risk factors (**Figure 4**). The validation cohort was used to assess the efficiency of the model. The initial 70:30 split yielded a C-index of 0.689 (95% CI: 0.671–0.707) in the training cohort and 0.689 (95% CI: 0.660–0.718) in the validation cohort. To address overfitting concerns, 1000 bootstrap resampling produced a bias-corrected C-index of 0.650. It indicates that the model had good discriminatory power. In the training cohort, the AUC of predicted nomogram for 6-months 1-year, 2-year and 3-year were 0.769 (0.738-0.799), 0.743 (0.708-0.777), 0.728 (0.685-0.771) and 0.743 (0.687-0.799) (**Figure 5A**). In the validation cohort, the AUC of predicted nomogram for 6-months 1-year, 2-year and 3-year were 0.755 (0.707-0.802), 0.722 (0.666-0.777), 0.727 (0.664-0.790) and 0.795 (0.726-0.863) (**Figure 5B**). The calibration plots for the training cohort predicting OS demonstrated a strong agreement between observed outcomes and model predictions (**Figure 5C**). Likewise, the calibration plots of the nomogram for predicting OS in the validation cohort also indicated a high level of accuracy (**Figure 5D**). The DCA demonstrated that the nomogram exhibited superior clinical utility, confirming its robust clinical value in both the training and validation cohorts (**Figure 6**). Additionally, a risk stratification system based on the total nomogram score was established to categorize patients into two groups: low-risk and high-risk. Notably, in the overall cohorts, patients with BM in the high-risk group exhibited significantly shorter OS compared to those in the low-risk and medium-risk group (**Figure 7**).

### 3.4 Visualization of feature importance in influencing OS

**Figure 8:**
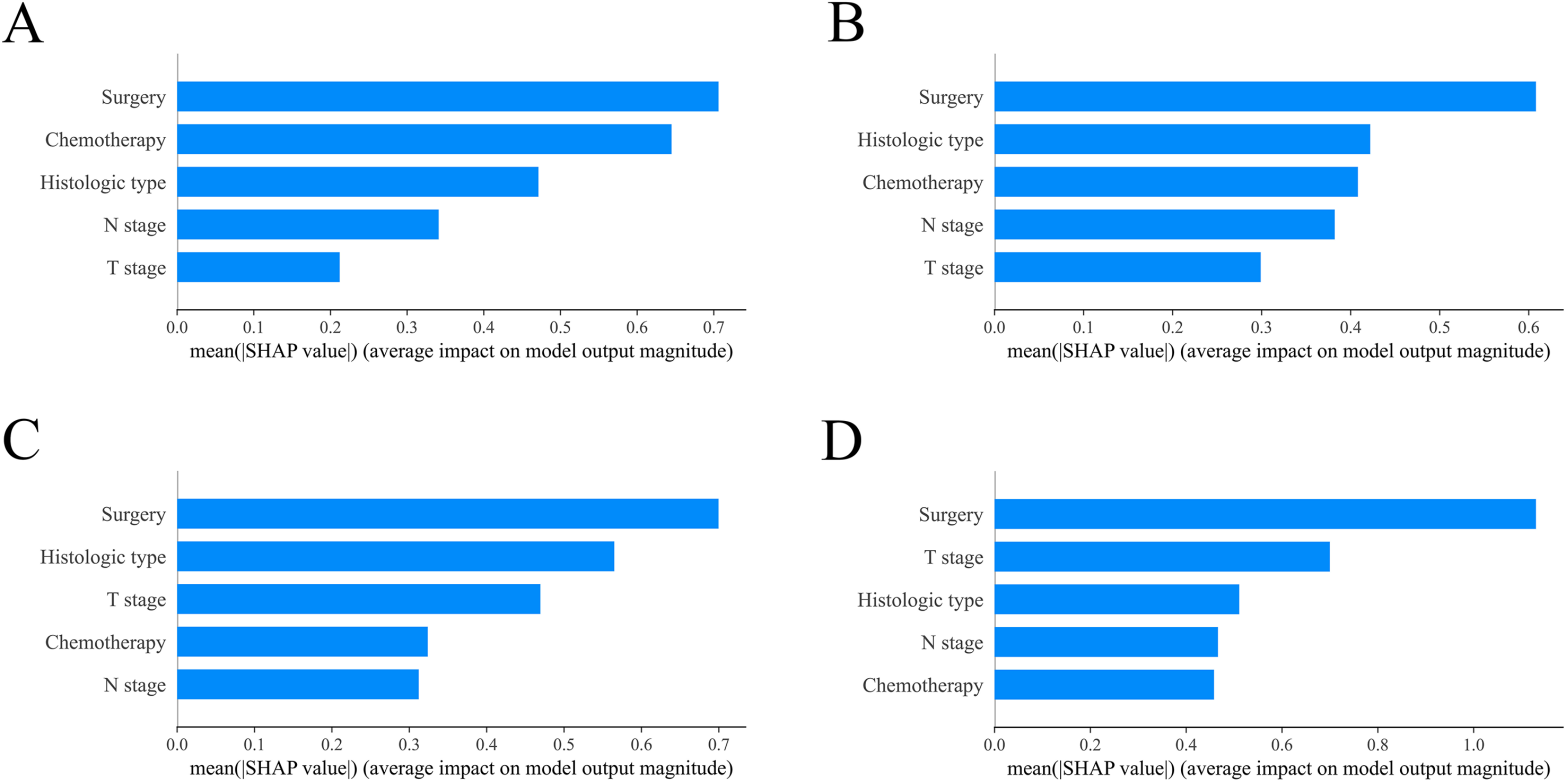
Model interpretation using SHAP (SHapley Additive exPlanations). The importance ranking of clinical characteristics in the XGBoost prognostic model is shown for different timeframes: **(A)** 6-month, **(B)** 1-year, **(C)** 2-year and **(D)** 3-year models. XGBoost: extreme Gradient Boosting.

After fitting the XGBoost model, we used SHAP summary plots to show the impact of five features on predicting OS for RCC patients with BM. **Figure 8** illustrates the relative influence of predictive features on model outcomes through a descending-ordered bar plot of mean absolute SHAP values, where larger SHAP magnitudes indicate stronger prognostic impact of the corresponding feature. Interestingly, the importance of SHAP plot features revealed that surgery was the most critical factor in the XGBoost model for predicting OS at 6-months, 1 year, 2 years, and 3 years.

### 3.5 Benefits of surgical treatment in RCC patients with BM across different subgroups

As shown in **Table 2**, there were significant differences in age, marital status, histologic type, grade, T stage, N stage, radiation, tumor size, bone metastases, liver metastases and lung metastases between the surgical and non-surgical groups (P < 0.05). To minimize potential confounding variables, a 1:1 PSM method was applied. After matching, 230 surgical patients and an equal number of non-surgical patients (460 patients in total) were successfully included. Notably, the baseline characteristics between the two groups were well-balanced (**Table 3**), with no significant differences observed (P > 0.05).

**Table 2:** Comparison of baseline variables between the non-surgical and radical nephrectomy groups before PSM.

Characteristics	Before PSM		P-value
	Non-Surgery (N = 1,043)	Surgery (N = 334)	
Age (years)			<0.001
<40	15 (1.44%)	10 (2.99%)	
40-49	89 (8.53%)	47 (14.07%)	
50-59	311 (29.82%)	108 (32.34%)	
60-69	387 (37.10%)	118 (35.33%)	
70-79	187 (17.93%)	46 (13.77%)	
80+	54 (5.18%)	5 (1.50%)	
Sex			0.736
Male	709 (67.98%)	231 (69.16%)	
Female	334 (32.02%)	103 (30.84%)	
Race			0.133
White	894 (85.71%)	289 (86.53%)	
Black	75 (7.19%)	15 (4.49%)	
Other	74 (7.09%)	30 (8.98%)	
Marital status			<0.001
Married	592 (56.76%)	225 (67.37%)	
Single	237 (22.72%)	47 (14.07%)	
S/D/W	214 (20.52%)	62 (18.56%)	
Histologic Type			<0.001
ccRCC	457 (43.82%)	227 (67.96%)	
pRCC	25 (2.40%)	10 (2.99%)	
Other	561 (53.79%)	97 (29.04%)	
Grade			<0.001
I	23 (2.21%)	4 (1.20%)	
II	89 (8.53%)	38 (11.38%)	
III	112 (10.74%)	120 (35.93%)	
IV	39 (3.74%)	117 (35.03%)	
Unknown	780 (74.78%)	55 (16.47%)	
Laterality			0.403
Left	517 (49.57%)	175 (52.40%)	
Right	526 (50.43%)	159 (47.60%)	
T stage			<0.001
T1	269 (25.79%)	32 (9.58%)	
T2	390 (37.39%)	57 (17.07%)	
T3	267 (25.60%)	214 (64.07%)	
T4	117 (11.22%)	31 (9.28%)	
N stage			0.005
N0	707 (67.79%)	254 (76.05%)	
N1	336 (32.21%)	80 (23.95%)	
Radiation			0.009
No	293 (28.09%)	69 (20.66%)	
Yes	750 (71.91%)	265 (79.34%)	
Chemotherapy			0.491
No	543 (52.06%)	166 (49.70%)	
Yes	500 (47.94%)	168 (50.30%)	
Size (mm)	89.00 (66.00, 110.00)	90.50 (72.00, 116.50)	0.007
Months from diagnosis to therapy			0.960
0 month	821 (78.72%)	264 (79.04%)	
≥ 1 month	222 (21.28%)	70 (20.96%)	
Bone metastases			<0.001
No	578 (55.42%)	229 (68.56%)	
Yes	465 (44.58%)	105 (31.44%)	
Liver metastases			<0.001
No	811 (77.76%)	300 (89.82%)	
Yes	232 (22.24%)	34 (10.18%)	
Lung metastases			<0.001
No	256 (24.54%)	133 (39.82%)	
Yes	787 (75.46%)	201 (60.18%)	
Median household income			0.708
< 50,000$	89 (8.53%)	22 (6.59%)	
50,000–69,999$	324 (31.06%)	103 (30.84%)	
70,000–89,999$	406 (38.93%)	135 (40.42%)	
90,000$ +	224 (21.48%)	74 (22.16%)	

**Table 3:** Comparison of baseline variables between the non-surgical and radical nephrectomy groups after PSM.

Characteristics	After PSM		P-value
	Non-Surgery (N = 230)	Surgery (N = 230)	
Age (years)			0.869
<40	5 (2.17%)	7 (3.04%)	
40-49	22 (9.57%)	29 (12.61%)	
50-59	70 (30.43%)	69 (30.00%)	
60-69	84 (36.52%)	83 (36.09%)	
70-79	44 (19.13%)	38 (16.52%)	
80+	5 (2.17%)	4 (1.74%)	
Sex			0.362
Male	165 (71.74%)	155 (67.39%)	
Female	65 (28.26%)	75 (32.61%)	
Race			0.863
White	203 (88.26%)	200 (86.96%)	
Black	12 (5.22%)	12 (5.22%)	
Other	15 (6.52%)	18 (7.83%)	
Marital status			0.924
Married	153 (66.52%)	149 (64.78%)	
Single	31 (13.48%)	33 (14.35%)	
S/D/W	46 (20.00%)	48 (20.87%)	
Histologic Type			0.761
ccRCC	150 (65.22%)	149 (64.78%)	
pRCC	12 (5.22%)	9 (3.91%)	
Other	68 (29.57%)	72 (31.30%)	
Grade			0.184
I	5 (2.17%)	4 (1.74%)	
II	43 (18.70%)	36 (15.65%)	
III	75 (32.61%)	80 (34.78%)	
IV	37 (16.09%)	55 (23.91%)	
Unknown	70 (30.43%)	55 (23.91%)	
Laterality			0.926
Left	117 (50.87%)	119 (51.74%)	
Right	113 (49.13%)	111 (48.26%)	
T stage			0.446
T1	42 (18.26%)	30 (13.04%)	
T2	49 (21.30%)	54 (23.48%)	
T3	113 (49.13%)	122 (53.04%)	
T4	26 (11.30%)	24 (10.43%)	
N stage			0.917
N0	167 (72.61%)	165 (71.74%)	
N1	63 (27.39%)	65 (28.26%)	
Radiation			0.824
No	54 (23.48%)	51 (22.17%)	
Yes	176 (76.52%)	179 (77.83%)	
Chemotherapy			1.000
No	115 (50.00%)	115 (50.00%)	
Yes	115 (50.00%)	115 (50.00%)	
Size (mm)	88.25 (65.00, 109.50)	89.57 (70.00, 115.00)	0.243
Months from diagnosis to therapy			1.000
0 month	181 (78.70%)	180 (78.26%)	
≥ 1 month	49 (21.30%)	50 (21.74%)	
Bone metastases			0.100
No	136 (59.13%)	154 (66.96%)	
Yes	94 (40.87%)	76 (33.04%)	
Liver metastases			1.000
No	203 (88.26%)	203 (88.26%)	
Yes	27 (11.74%)	27 (11.74%)	
Lung metastases			0.561
No	80 (34.78)	87 (37.83%)	
Yes	150 (65.22)	143 (62.17%)	
Median household income			0.140
< 50,000$	18 (7.83%)	15 (6.52%)	
50,000–69,999$	61 (26.52%)	77 (33.48%)	
70,000–89,999$	108 (46.96%)	86 (37.39%)	
90,000$ +	43 (18.70%)	52 (22.61%)	

**Figure 9:**
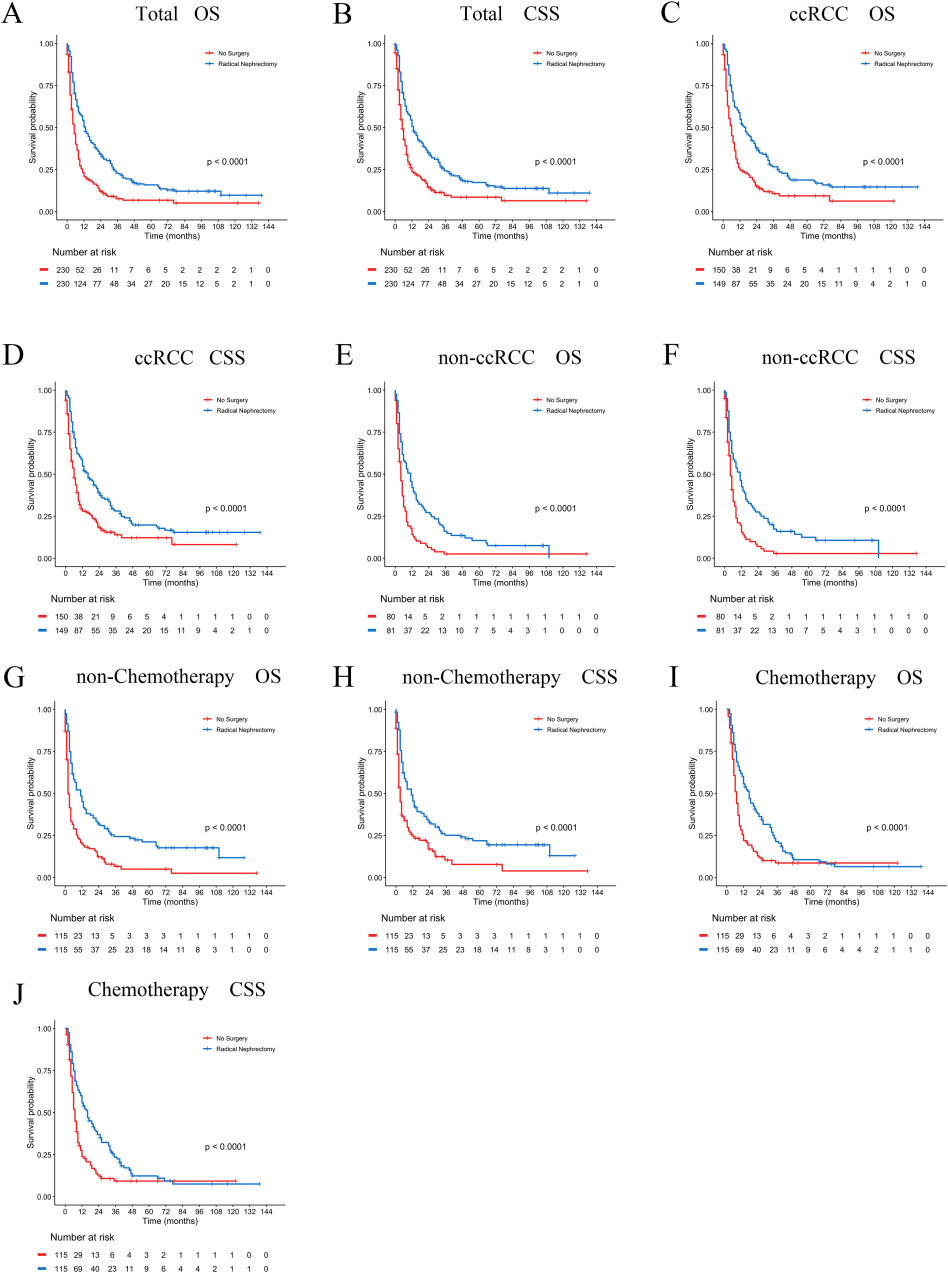
PSM-adjusted OS and CSS in brain metastatic RCC patients undergoing surgical treatment. Kaplan–Meier (K–M) survival analysis: **(A)** OS of all RCC patients with BM; **(B)** CSS of all RCC patients with BM; **(C)** OS of RCC patients with BM in the ccRCC subgroup; **(D)** CSS of RCC patients with BM in the ccRCC subgroup; **(E)** OS of RCC patients with BM in the non-ccRCC subgroup; **(F)** CSS of RCC patients with BM in the non-ccRCC subgroup; **(G)** OS of RCC patients with BM not receiving chemotherapy; **(H)** CSS of RCC patients with BM not receiving chemotherapy; **(I)** OS of RCC patients with BM receiving chemotherapy; **(J)** CSS of RCC patients with BM receiving chemotherapy.

In the PSM-adjusted cohort, the surgical group demonstrated a 48.4% reduction in overall mortality risk (P < 0.001, HR: 0.516; 95% CI: 0.423–0.630) (**Figure 9A**) and approximately a 45.4% reduction in BM-related mortality risk (P < 0.001, HR: 0.546; 95% CI: 0.444–0.670) (**Figure 9B**). According to the stratified Kaplan-Meier survival analysis, OS and CSS of patients with BM in the entire cohort, ccRCC subgroup, non-ccRCC subgroup, non-chemotherapy group, and chemotherapy group were significantly improved after undergoing radical nephrectomy, leading to a notable extension of patient survival (**Figure 9C–J**).

Furthermore, we conducted subgroup analyses to evaluate potential interaction effects, with adjustments for relevant covariates. As shown in **Supplementary Table S1** (**Supplementary File 1**), radical nephrectomy provided significant survival benefits (all P<0.05) for most BM patients except: age extremes (<50 or >70 years), black race, low-grade tumors (Grade I), and patients with liver metastases. Multivariable-adjusted interaction tests revealed no significant subgroup heterogeneity (all interaction P>0.05), confirming the general applicability of surgical benefits in the majority of patients.

## 4 Discussion

This study utilized data from the SEER database to construct a predictive model for OS in RCC patients with BM, addressing some of the limitations present in previous research (25). The XGBoost and RF algorithms were innovatively employed for the preliminary selection of features, followed by multivariate Cox regression analysis to control for potential confounding effects among the variables. We subsequently developed a nomogram model incorporating five variables, which demonstrated strong performance in the validation cohort and revealed its substantial clinical applicability. In addition, we constructed a feature importance ranking using SHAP values to identify variables that influenced the prognosis. Our analysis indicated that surgical treatment was the most important prognostic risk factor for OS at 6-months, 1-year, 2-years, and 3-years, with patients who underwent surgery showing better survival at each time point compared to those who did not undergo surgery.

While cytoreductive nephrectomy (CN) was historically considered standard for metastatic RCC, its role in the targeted therapy era remains debated (26, 27). Existing evidence on CN primarily derives from non-BM cohorts (28, 29), leaving its utility in RCC patients with BM unaddressed. Furthermore, the CARMENA trial in patients with mRCC indicated that sunitinib monotherapy was non-inferior to the combined approach of nephrectomy plus sunitinib in the majority of patients, as evidenced by a hazard ratio of 0.97 (95% confidence interval: 0.79-1.19; p = 0.8) (27).

In contrast to the aforementioned findings, a large-scale retrospective study encompassing 15,390 mRCC patients receiving targeted therapies confirmed significantly prolonged median OS in the CN-treated group (17.1 months) compared to the non-CN group (7.7 months), with the CN cohort maintaining superior prognostic outcomes even after PSM adjustment for baseline characteristics (30). Bhindi et al. observed that CN remains clinically beneficial for patients with limited metastatic burden, carefully selected patient subgroups, and those exhibiting favorable responses to initial systemic therapy (31). A separate IMDC-based study demonstrated that both therapeutic sequences incorporating CN and sunitinib—specifically primary CN with subsequent sunitinib versus sunitinib pretreatment followed by CN—yielded significantly superior median OS outcomes relative to sunitinib monotherapy (32). Our PSM-adjusted analysis specifically in BM patients demonstrates that CN confers a 48.4% reduction in mortality risk (HR: 0.516), supporting its selective utility in this high-risk subgroup. Moreover, Takemura et al. analyzed data from the IMDC and found that, among select mRCC patients receiving frontline immuno-oncology-based combination therapies, the addition of CN was associated with a survival benefit (33). Other studies had also substantiated that CN could provide extended survival time for mRCC patients (34–36). Our study observed an association between CN and improved prognosis in RCC patients with BM, consistent with previous observational studies (25). However, causality cannot be definitively established due to potential residual confounding.

Currently, the relationship between RCC histological subtypes and prognosis in patients with BM remains inconclusive. In a cohort study of 325 mRCC patients, researchers pointed out that non-ccRCC patients exhibited significantly worse progression-free survival (PFS) and OS compared to their ccRCC counterparts (37). This finding was further supported by Delahunt et al., who reported that ccRCC was associated with improved survival outcomes, while collecting duct RCC and undifferentiated RCC presented the most unfavorable prognosis (38). Additionally, Luo et al. substantiated these observations in mRCC patients, revealing that ccRCC was correlated with superior OS and CSS (39). These findings align with multiple studies consistently indicating better survival rates associated with the ccRCC subtype (19, 40). In line with these established patterns, our study findings revealed that patients with BM originating from non-ccRCC histological subtypes had a significantly poorer prognosis compared to those with ccRCC, thereby corroborating previous research outcomes.

The TNM staging system is currently the most widely adopted international framework for tumor classification and serves as the standard methodology for clinical staging of malignant neoplasms. Within the TNM staging system, the T category denotes the primary tumor’s size, depth of invasion, and anatomical extent, while the N category indicates the regional lymph node involvement in terms of location and number of metastatic nodes, with higher T and N categories being associated with increased probability of distant metastasis (41, 42). Unexpectedly, we observed a strong correlation between T1 stage tumors and a poorer prognosis, which contrasts with previous large population studies on RCC metastasizing to other organs, where higher T stage mRCC was associated with a worse prognosis (19, 40, 43, 44). This paradoxical finding may be attributed to several factors: data artifacts: potential misclassification of T-stage in the SEER database may introduce bias into survival association analyses; biological characteristics: T1 tumors presenting with early BM may represent a distinct subgroup harboring aggressive molecular features such as sarcomatoid differentiation or CDKN2A/B loss; treatment disparities: patients with higher T-stage (T2-T4) are more likely to receive multimodal therapies, potentially diluting the independent prognostic value of T-stage; residual confounding: unmeasured clinical factors may exert confounding effects on survival outcomes (31, 45, 46). The higher N-stage being indicative of a poorer prognosis is consistent with prior research. Although chemotherapy showed limited efficacy in the treatment of RCC, our study revealed that it remained one of the primary therapeutic modalities for a substantial proportion of patients with mRCC, demonstrating a significant improvement in OS (47). Additionally, emerging evidence from independent studies had clarified that chemotherapy administration was associated with improved OS and CSS in RCC patients with bone metastases (48).

Despite rigorous adjustments through multivariate Cox regression and PSM, our findings may still be influenced by residual confounding from unmeasured selection biases. Patients undergoing surgery may have had intrinsic advantages that were not adequately captured in the available data. These latent factors could partially explain the observed survival benefit, indicating that the association between surgery and improved outcomes requires cautious interpretation. To translate these findings into clinical practice, the developed nomogram provides clinically actionable support for RCC patients with BM by: quantifying surgical benefit-risk ratios during multidisciplinary preoperative evaluations (e.g., favoring intervention when predicted survival exceeds 50%); screening potential clinical trial candidates (e.g., excluding high-risk patients with <3-month predicted survival); enabling rapid risk stratification in primary care settings using basic parameters (T/N stage, histology); and visualizing outcome differences for treatment decision-making. Compared to existing models, it demonstrates superior dynamic time-dependent prediction (6-month to 3-year intervals), BM-specific optimization (incorporating neurosurgical/radiotherapy variables), and SHAP-enhanced interpretability.

We acknowledge several limitations in our study. Firstly, the sample size was relatively small, and the imputation of missing data using ML algorithms may introduce discrepancies compared to real-world data. Secondly, the SEER database lacks detailed information on treatment regimens and follow-up data (including tumor recurrence and progression) for RCC patients with BM, which poses challenges for more comprehensive prognostic analysis. Thirdly, the SEER database does not capture key prognostic variables such as MDC risk stratification parameters (e.g., hemoglobin, serum calcium, LDH), comorbidity indices (Charlson score), performance status (KPS/ECOG), detailed BM characteristics (number, location, hemorrhage status), neurological symptoms, or other laboratory markers, potentially limiting the model’s capacity to capture clinical complexity (49–52). Prospective validation in enriched cohorts is needed to refine this nomogram’s utility for guiding multimodal therapy in RCC patients with BM. Finally, to further address confounding, future studies should employ advanced causal inference methods, such as inverse probability treatment weighting (IPTW), instrumental variable analysis, or target trial emulation.

## 5 Conclusion

This study presents a novel nomogram specifically designed for RCC patients with BM, incorporating machine learning-based feature selection (XGBoost/RF), SHAP interpretability analysis, and PSM-validated surgical outcomes. The nomogram exhibited superior predictive performance in the training and validation cohorts. Through SHAP value analysis, surgical intervention was identified as the most critical factor influencing OS. The nomogram specifically addresses the clinical challenge of identifying BM patients who are most likely to benefit from surgery, while also providing clinically actionable thresholds to guide multidisciplinary decision-making.

## Data Availability

The datasets presented in this study can be found in online repositories. The names of the repository/repositories and accession number(s) can be found in the article/**Supplementary Material**.
