# Supplementary material for: A novel nomogram for survival prediction in renal cell carcinoma patients with brain metastases: an analysis of the SEER database
Source: Front Immunol. 2025 Jun 30;16:1572580. doi: 10.3389/fimmu.2025.1572580 (PMC12256520; doi:10.3389/fimmu.2025.1572580)
Supplement: Supplementary file 1 [file DataSheet1.docx]

**Table S1** Subgroup analysis for interaction between non-surgical and radical nephrectomy groups in overall survival for renal cell carcinoma patients with brain metastases.

| Subgroup | Count | Percent (%) | HR (95% CI) | P value | P for interaction |
| --- | --- | --- | --- | --- | --- |
| Age (years) |  |  |  |  | 0.064 |
| <40 | 12 | 2.6 | 0.81 (0.23-2.88) | 0.750 |  |
| 40-49 | 51 | 11.1 | 0.71 (0.39-1.32) | 0.281 |  |
| 50-59 | 139 | 30.2 | 0.44 (0.30-0.64) | <0.001 |  |
| 60-69 | 167 | 36.3 | 0.47 (0.34-0.66) | <0.001 |  |
| 70-79 | 82 | 17.8 | 0.68 (0.42-1.08) | 0.099 |  |
| 80+ | 9 | 2 | / | / |  |
| Sex |  |  |  |  | 0.905 |
| Male | 320 | 69.6 | 0.51 (0.40-0.65) | <0.001 |  |
| Female | 140 | 30.4 | 0.49 (0.35-0.71) | <0.001 |  |
| Race |  |  |  |  | 0.440 |
| White | 403 | 87.6 | 0.51 (0.41-0.63) | <0.001 |  |
| Black | 24 | 5.2 | 0.86 (0.34-2.17) | 0.749 |  |
| Other | 33 | 7.2 | 0.33 (0.15-0.76) | 0.009 |  |
| Marital status |  |  |  |  | 0.532 |
| Married | 302 | 65.7 | 0.56 (0.43-0.71) | <0.001 |  |
| Single | 64 | 13.9 | 0.52 (0.30-0.91) | 0.023 |  |
| S/D/W | 94 | 20.4 | 0.36 (0.23-0.57) | <0.001 |  |
| Histologic Type |  |  |  |  | 0.962 |
| ccRCC | 299 | 65 | 0.52 (0.40-0.67) | <0.001 |  |
| pRCC | 21 | 4.6 | 0.26 (0.08-0.87) | 0.028 |  |
| Other | 140 | 30.4 | 0.51 (0.36-0.72) | <0.001 |  |
| Grade |  |  |  |  | 0.777 |
| I | 9 | 2 | 0.42 (0.09-1.97) | 0.271 |  |
| II | 79 | 17.2 | 0.50 (0.30-0.84) | 0.009 |  |
| III | 155 | 33.7 | 0.40 (0.28-0.56) | <0.001 |  |
| IV | 92 | 20 | 0.44 (0.28-0.68) | <0.001 |  |
| Unknown | 125 | 27.2 | 0.61 (0.41-0.91) | 0.015 |  |
| Laterality |  |  |  |  | 0.066 |
| Left | 236 | 51.3 | 0.62 (0.47-0.81) | 0.001 |  |
| Right | 224 | 48.7 | 0.43 (0.32-0.57) | <0.001 |  |
| AJCC T stage |  |  |  |  | 0.077 |
| T1 | 72 | 15.7 | 0.36 (0.21-0.61) | <0.001 |  |
| T2 | 103 | 22.4 | 0.39 (0.25-0.59) | <0.001 |  |
| T3 | 235 | 51.1 | 0.69 (0.52-0.91) | 0.008 |  |
| T4 | 50 | 10.9 | 0.45 (0.25-0.81) | 0.008 |  |
| AJCC N stage |  |  |  |  | 0.482 |
| N0 | 332 | 72.2 | 0.49 (0.39-0.62) | <0.001 |  |
| N1 | 128 | 27.8 | 0.56 (0.39-0.81) | 0.002 |  |
| Radiation |  |  |  |  | 0.689 |
| No | 105 | 22.8 | 0.54 (0.36-0.83) | 0.005 |  |
| Yes | 355 | 77.2 | 0.51 (0.41-0.64) | <0.001 |  |
| Chemotherapy |  |  |  |  | 0.040 |
| No | 230 | 50 | 0.46 (0.35-0.62) | <0.001 |  |
| Yes | 230 | 50 | 0.55 (0.42-0.73) | <0.001 |  |
| Months from diagnosis to therapy |  |  |  |  | 0.390 |
| 0 month | 361 | 78.5 | 0.51 (0.41-0.63) | <0.001 |  |
| ≥ 1 month | 99 | 21.5 | 0.52 (0.33-0.81) | 0.004 |  |
| Bone metastases |  |  |  |  | 0.629 |
| No | 290 | 63 | 0.52 (0.40-0.67) | <0.001 |  |
| Yes | 170 | 37 | 0.54 (0.39-0.75) | <0.001 |  |
| Liver metastases |  |  |  |  | 0.344 |
| No | 406 | 88.3 | 0.50 (0.4-0.62) | <0.001 |  |
| Yes | 54 | 11.7 | 0.71 (0.4-1.25) | 0.232 |  |
| Lung metastases |  |  |  |  | 0.117 |
| No | 167 | 36.3 | 0.41 (0.29-0.58) | <0.001 |  |
| Yes | 293 | 63.7 | 0.60 (0.47-0.76) | <0.001 |  |
| Median household income |  |  |  |  | 0.512 |
| < 50,000$ | 33 | 7.2 | 0.45 (0.21-0.97) | 0.041 |  |
| 50,000–69,999$ | 138 | 30 | 0.61 (0.43-0.88) | 0.008 |  |
| 70,000–89,999$ | 194 | 42.2 | 0.45 (0.33-0.62) | <0.001 |  |
| 90,000$ + | 95 | 20.7 | 0.54 (0.35-0.84) | 0.007 |  |

D/S/W, divorced/separated/widowed; ccRCC, clear cell renal cell carcinoma; pRCC, papillary renal cell carcinoma.
